# Task-Decomposed Language Supervision for End-to-End Autonomous Driving

**DOI:** 10.3390/s26206455

**Published:** 2026-10-12

**Authors:** Jiwon Lee, Seok-Cheol Kee

**Affiliations:** Department of Intelligent Systems and Robotics, Chungbuk National University, Cheongju 28644, Republic of Korea; a1038562@chungbuk.ac.kr

**Keywords:** end-to-end autonomous driving, large language models, language supervision, trajectory planning, nuScenes, representation transfer

## Abstract

End-to-end (E2E) autonomous driving integrates perception, prediction, and planning, yet often lacks explicit supervision for high-level driving semantics. We propose Task-Decomposed Language Supervision (TaLS), a training-only framework that organizes DriveLM question–answer annotations into four sequential stages: Object/Scene, Motion, Risk, and Planning. During training, the answer-end hidden state of each stage is projected into designated driving-task queries through gate-free residual injection. The autoregressive language model and injections are removed at inference, while the BEV token modules remain active. On the nuScenes open-loop benchmark, the TaLS reference run reduces the TemAvg box-collision metric by 31.3% relative to a matched GenAD baseline with comparable trajectory L2 and perception performance. Across three seeds, TemAvg improvements occur in two runs, with closely matched mean collision rates; under NoAvg, TaLS achieves an equal or lower NoAvg collision rate in all three runs. Component ablations identify stage-wise injection and decomposition as contributors to the observed collision reduction, and a pruning analysis separates the roles of retained BEV token modules and training-time language supervision. TaLS also maintains a lower absolute collision metric under four tested camera degradations. These findings illustrate how structured language supervision can shape open-loop planning behavior without autoregressive language-model inference.

## 1. Introduction

End-to-end models for autonomous driving build a bird’s-eye-view (BEV) representation from multi-camera images and learn object detection, map perception, motion prediction, and ego planning within a single network. UniAD [1], VAD [2], and GenAD [3] showed that such integration reduces error accumulation relative to modular pipelines and improves planning performance. In particular, GenAD formulates future trajectories as a generation problem and achieves strong planning performance through instance-centric representations and latent-variable trajectory generation.

However, these models encode the scene primarily through geometric and motion cues, and do not explicitly represent high-level semantic reasoning such as “which object is dangerous” or “what is a safe driving behavior.” To address this, research combining LLMs/VLMs has emerged, falling into two categories. (i) Inference-time LLM-driven methods (DriveVLM [4], LMDrive [5], etc.) are highly expressive but challenging for real-time deployment due to the cost of autoregressive generation. (ii) Training-time language-supervision methods (VLP [6], VLM-AD [7], DiMA [8], etc.) remove the LLM at inference and are therefore efficient, but they generally transfer language-derived knowledge through feature alignment, auxiliary semantic targets, or cross-modal distillation rather than directly coupling stage-specific reasoning states to the corresponding task queries.

The two families above expose a design tension that motivates this work. Inference-time methods keep the language model in the loop and can therefore reason explicitly about a scene, but the cost of autoregressive decoding can be difficult to reconcile with the update rates required for closed-loop control, and the additional memory footprint of the language model competes with the perception stack for on-board resources. Training-time supervision methods remove this cost at inference by transferring language-derived knowledge to the driving model during training. Existing approaches employ various mechanisms, including feature alignment, auxiliary semantic targets, and cross-modal distillation at different levels of the driving stack. Although effective, these methods generally use language representations as supervision targets or auxiliary signals rather than directly incorporating stage-specific reasoning states into the task queries that execute the corresponding driving subtasks. This distinction motivates a closer coupling between the structure of language reasoning and the query-based task hierarchy of an E2E driving model.

This motivates two design choices in TaLS. First, rather than using language representations only as alignment targets or auxiliary supervision, we inject the representation of each reasoning stage directly into the query representation used by the corresponding driving component. Second, we preserve the sequential structure of the reasoning process—perception, motion, risk, and planning—so that later language states are conditioned on earlier stages before being delivered to the associated task queries. TaLS therefore focuses not merely on transferring language knowledge, but on coupling task-decomposed reasoning states with the query-based computation of the E2E driving stack.

This paper belongs to category (ii). While recent work also supervises across pipeline stages (e.g., VERDI [9]), TaLS differs in the specific supervision mechanism and in its analysis of that mechanism. We restructure the DriveLM QA into a four-stage chain (perception → motion → risk → planning) (TD-QA) and, during training, inject the LLM hidden state at each stage’s answer position directly into the corresponding task decoder’s queries as a gate-free residual. This aligns the task hierarchy of the E2E model (detection → prediction → planning) with the stages of language reasoning in a stage-aligned manner. After training, the autoregressive LLM is removed, and the inference graph retains only the BEV Q-Former and the BEV token augmentation modules. We further analyze, on the trained model, which planning gains persist in the retained weights after the language branch is removed. All planning results are measured under the standard nuScenes open-loop protocol, which isolates planning behavior against recorded expert trajectories in a controlled and reproducible setting; extending this evaluation to closed-loop driving is left to future work (Section 4.6).

**Hidden-state injection versus language distillation.** Conventional distillation [10] uses a teacher representation as an auxiliary target. TaLS instead places each stage hidden state directly in the driving forward path as an additive decoder-query residual, so the driving objectives update the task decoders under the injected context. Section 3.4 defines this mechanism, and the controlled ablations in Section 4.4 separate it from the auxiliary distillation objective.

**TD-QA versus generic chain-of-thought supervision.** A chain-of-thought (CoT) [11] trace supervises a single output—the planner, or a language head—with a linear sequence of reasoning steps, and the steps are consumed only through their effect on that one output. TD-QA differs in that the reasoning chain is aligned with the task hierarchy of the driving network through designated task-query injection pathways: the Object/Scene stage conditions the detection queries, the Motion stage conditions the motion queries, and the Risk and Planning stages jointly condition the ego query through separate stage-specific projections. The decomposition is therefore not only a prompting device but also a structured supervision mechanism that establishes explicit stage-to-query correspondence while allowing later reasoning stages to be conditioned on earlier ones. We quantify what this structure contributes, relative to undecomposed language supervision, in Section 4.4.

The contributions of this paper are as follows.

- **TD-QA (task-decomposed question answering).** We construct a four-stage reasoning chain from driving QA annotations, aligned with the task hierarchy of the E2E driving model. During training, the LLM hidden state of each stage is injected into its designated task-query pathway.
- **LLM-free inference.** The 1.5B-scale LLM and the stage-wise injection are used only during training; at inference, the autoregressive LLM is removed, so the model operates without autoregressive generation cost.
- **Mechanism analysis.** Using a pruning ablation on the trained model, we analyze which L2 and collision gains depend on the retained BEV token path (Q-Former, SLG, Fusion) and which persist after this path is disabled. Controlled component ablations further separate the contribution of the stage-wise injection from that of the auxiliary distillation objective.
- **Robustness and optimization analysis.** We report TD-QA construction statistics, active inference-parameter tracing, phase-wise training diagnostics, and zero-shot evaluation under four camera degradations to characterize optimization behavior and robustness of the proposed training-only supervision.
- **Reproducibility, semantic control, and deployment cost.** We add a three-seed analysis of GenAD and TaLS, two fixed stage-mismatch controls that probe the stage-to-task routing under distinct non-aligned permutations, and matched A100 measurements of active parameters, estimated FLOPs, latency, and peak GPU memory for the LLM-free inference graph.

## 2. Related Work

**End-to-end autonomous driving.** UniAD [1] unified multiple tasks in a query-based manner; VAD [2] improved efficiency with a vectorized scene representation; GenAD [3] treated future trajectories as a generation problem and raised planning performance via instance-centric interaction and latent-variable generation. This work adopts GenAD as the base model and adds language supervision on top of it.

**Open-loop evaluation on nuScenes.** The planning results discussed above are obtained under open-loop evaluation, in which the planned trajectory is compared against a recorded human trajectory without executing it. Two studies have highlighted important limitations of this open-loop protocol. Zhai et al. [12] reported that a multi-layer perceptron taking only the ego vehicle’s past trajectory and velocity as input, without any camera or LiDAR perception, attains planning scores comparable to contemporary end-to-end systems, indicating that the benchmark rewards extrapolation of ego motion. Li et al. [13] reached a similar conclusion and showed that supplying the ego status dominates the reported metrics; consistent with this, VAD [2] and GenAD [3] report results without ego status, and we follow the same setting. A second and separate difficulty is that the reported numbers depend on how the metrics are aggregated over the predicted horizon: UniAD [1] evaluates the value at each endpoint, whereas ST-P3 [14] and VAD [2] average over all waypoints up to that horizon, so the same model can differ substantially between the two conventions. We therefore report both aggregation protocols explicitly (Section 3.7), providing a consistent basis for interpreting TaLS within the established nuScenes open-loop benchmark.

**LLMs/VLMs for driving—inference-time-driven.** DriveGPT4 [15], LMDrive [5], and DriveVLM(-Dual) [4] place the LLM/VLM inside the inference loop to gain explainability and generalization, but they incur autoregressive generation cost and additional inference overhead. SimpleLLM4AD [16] similarly decomposes driving into a multi-stage QA chain (perception, prediction, planning, behavior) connected as a graph, but reasons stage-by-stage with the VLM at inference. In contrast, TaLS uses its stage-wise QA only during training and removes the autoregressive LLM at inference. More broadly, recent work surveys the progression from LLM-based vehicular intelligence toward agentic and embodied AI systems, highlighting the growing role of language-conditioned reasoning in next-generation intelligent vehicles [17].

**LLMs/VLMs for driving—training-time supervision.** VLP [6] trains a vision-based planner jointly with a frozen language encoder, aligning agent-level BEV features and the ego planning query with text features derived from ground-truth labels through contrastive learning; VLM-AD [7] prompts a vision-language model to produce free-form reasoning and structured action annotations for each frame and supervises auxiliary heads attached to the ego feature with them; and DiMA [8] uses the vision-based planner as a tokenizer for a multimodal language model and distills features from the language model’s planning head into the planning transformer under a set of surrogate tasks. In all three, the language branch is discarded, or becomes optional, at inference. Closest to our setting, VERDI [9] aligns intermediate perception, prediction, and planning representations with VLM-generated reasoning features at the corresponding stages. These methods share the advantage of being LLM-free (or LLM-optional) at inference. TaLS also belongs to this category, but differs in two respects: (i) rather than aligning module outputs to generated explanation text or distilling a single global feature, TaLS injects the answer-end LLM hidden state of each reasoning stage directly into the corresponding decoder queries as a gate-free residual during training; and (ii) we provide a mechanistic analysis examining which planning gains persist in the retained decoder weights and which depend on the inference-time BEV token path (Section 3.5 and Section 4.4). Mechanistically, the distinction from stage-wise alignment methods such as VERDI is that TaLS places the answer-end language state directly in the forward computation of the task query before the driving objective is evaluated. Alignment or distillation constrains one representation to match another through an auxiliary objective, whereas the TaLS residual directly conditions the representation consumed by the Agent, Motion, or ego-planning computation on language-supervised frames. Consequently, the driving losses update the task decoders under the injected stage context; the w/o injection control in Section 4.4 tests this forward-path contribution separately from the auxiliary distillation loss.

Table 1 summarizes the closest language-based E2E driving approaches in terms of supervision structure, where the language signal enters the driving stack, and whether language-model computation remains at inference.

**Robustness under visual degradation.** Camera-based autonomous-driving models must preserve useful scene representations when image quality is degraded. SUSTechGAN [18], for example, studies rain- and night-condition image generation for improving object detection, illustrating the importance of adverse-condition perception. Complementing such perception-focused work, Section 4.5 evaluates the downstream planning behavior of TaLS without corruption-aware retraining under Gaussian noise, low light, motion blur, and single-camera dropout.

**Bridging visual features and language models.** Connecting a visual encoder to a frozen language model requires compressing dense features into a small number of tokens that the language model can consume. BLIP-2 [19] introduced the Querying Transformer, a set of learnable queries that cross-attend to image features and emit a fixed-length sequence in the embedding space of the language model. We adapt this design to the bird’s-eye-view domain (Section 3.2), using the resulting tokens both as the language-model prefix and as the input to the modules that remain at inference. DriveLM [20] provides the graph-structured driving question-answering annotations on nuScenes from which our four-stage chain is constructed.

## 3. Materials and Methods

### 3.1. Overview

**Figure 1:**
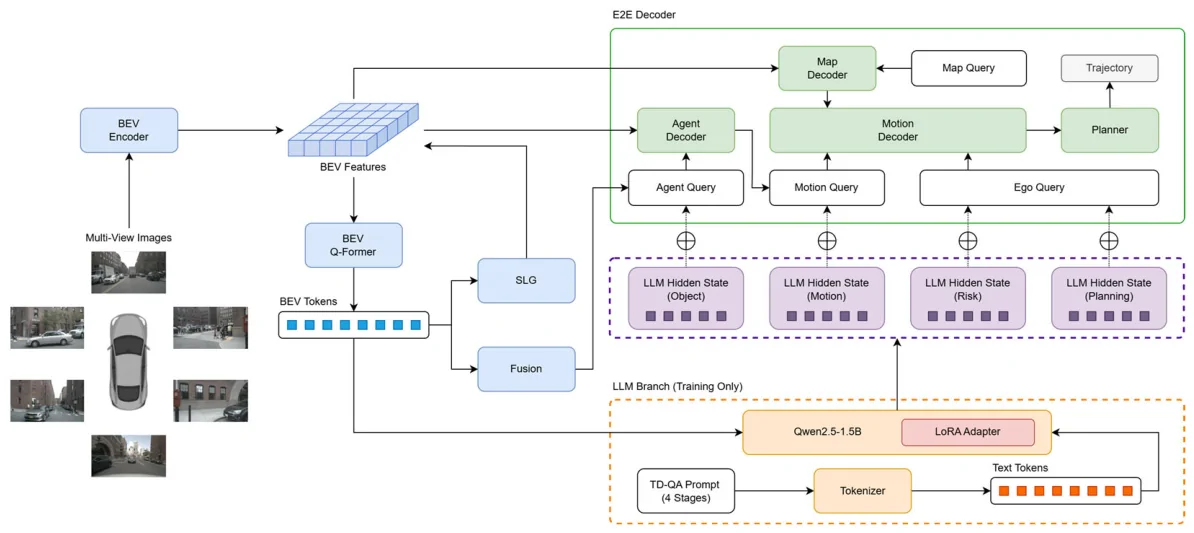
Overview of TaLS. Solid components are retained at inference; dashed components—the LLM branch (Qwen2.5-1.5B with a LoRA adapter, tokenizer, and text tokens) together with the four LLM hidden states and the stage-wise ⊕ injections—are used during training only and are removed at inference. Multi-view images are encoded by a frozen BEV encoder into BEV features, which drive the E2E decoder (Agent, Map, and Motion decoders and the Planner); the motion queries are derived from the Agent Decoder output. During training, the BEV Q-Former compresses the BEV features into BEV tokens that (i) prefix the Qwen input alongside the tokenized four-stage TD-QA prompt and (ii) feed two lightweight modules, SLG and Fusion. The answer-end hidden state of each reasoning stage is injected as a gate-free residual into the corresponding queries (Object→Agent, Motion→Motion, and both Risk→Ego and Planning→Ego); the ego query thus receives the risk and planning states. The BEV token path (BEV Q-Former, SLG, Fusion) is retained at inference: SLG adds a gated residual back into the BEV features, and Fusion augments the detection (Agent) queries. At inference, the autoregressive LLM and the injections are removed, so planning relies only on the E2E decoder and the retained BEV token path. Arrows denote the direction of feature or token flow; arrows leading to the ⊕ nodes indicate addition of stage-specific residuals to task queries.

We refer to the complete framework as TaLS (Task-Decomposed Language Supervision). Its core consists of task-decomposed question answering (TD-QA; Section 3.3) and stage-wise hidden-state injection (Section 3.4), which connects the language supervision to the task queries of the driving network. TaLS consists of a GenAD-based E2E decoder and an LLM branch (Figure 1). In the LLM branch, the autoregressive LLM and the stage-wise injection are used only during training (for the inference-time graph, see Section 3.5). Six surround-view camera images are converted by a ResNet-50 [21] backbone and a BEV encoder [22] into BEV features B ∈ ℝ^H×W×C^ (H = W = 100, C = 256). The E2E decoder follows the GenAD pipeline: Agent Decoder (object detection), Map Decoder (vectorized map), Motion Decoder (agent–ego interaction and agent/ego–map cross-attention), the generative trajectory module, and the Planner.

During training, BEV tokens—the BEV features compressed by the BEV Q-Former—are fed to a LoRA-based Qwen2.5-1.5B; the TD-QA reasoning chain is performed, and each stage’s hidden state is injected into the corresponding decoder. The BEV tokens are also fed back into the model through two lightweight modules: **SLG** (Spatial Language Grounding; BEV tokens are back-projected onto the BEV grid using the Q-Former’s cross-attention weights and added to the BEV as a gated residual) and **Fusion** (detection queries cross-attend to the BEV tokens and are augmented as a gated residual). As shown in the analysis of Section 3.5, although these two modules add only small residuals, they contribute to inference performance (especially L2) and are therefore retained in the inference graph.

### 3.2. BEV Q-Former

To compress the BEV features into a small number of tokens suitable for the language model input space, we adapt BLIP-2′s Q-Former [19] to the BEV domain. N_q_ = 32 learnable query tokens cross-attend, through a BERT structure (12 layers), to the spatially pooled and projected BEV tokens (25 × 25 = 625) to summarize the scene. The output is linearly projected to BEV tokens V ∈ ℝ^N^_q_^×d^_LLM_ in the LLM embedding dimension (d_LLM_ = 1536).

V = Q-Former(pool(B)) ∈ ℝ^32×1536^. (1)

### 3.3. Task-Decomposed Question Answering (TD-QA)

We restructure the DriveLM [20] driving QA into a four-stage reasoning chain based on keywords. The four stages and their corresponding tasks are summarized in Table 2.

TD-QA construction and coverage. DriveLM QA annotations are available for 4072 of the 28,130 training frames (14.5%), corresponding to 4072 language-supervised frames per epoch. Among the annotated frames, the source lists contain on average 39.9 perception QAs, 21.6 planning QAs, and 1.0 behavior QA per frame. Scene and Motion candidates are drawn from the DriveLM perception questions, whereas Risk and Planning candidates are drawn from the planning questions. The active matching keywords for each stage are as follows:

- Object/Scene: important/object/describe/what is/what are/scene/around/front/left/right
- Motion: moving/driving
- Risk: danger/collision/avoid/careful/caution
- Planning: safe action/should/behavior/ego vehicle should/what action

For every annotated frame, keyword matching produced at least one candidate for all four stages, so the random-fallback branch was never triggered and no source list was empty. When multiple candidates match a stage, one is selected with the training RNG controlled by the global seed, providing reproducible sampling under the same data pipeline while allowing the selected QA to vary across epochs. The DriveLM annotations are used without manual correction. Because keyword sets can overlap, the routing is treated as an automatic heuristic grouping rather than a manually curated semantic partition. Section 4.6 provides an exhaustive dataset-wide characterization of this routing procedure.

**Figure 2:**
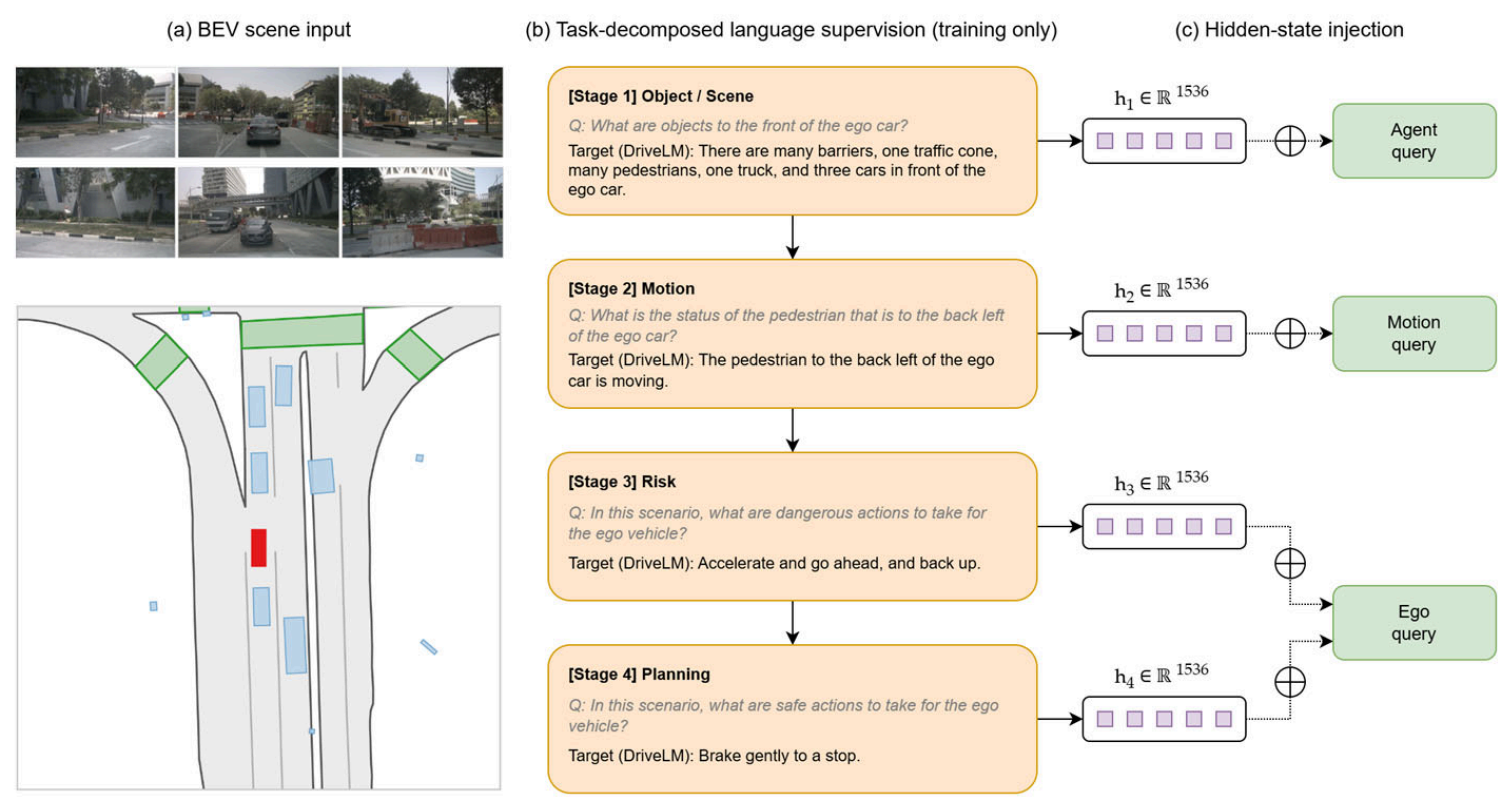
Task-decomposed question answering (TD-QA) and stage-wise hidden-state injection during training. (**a**) Input for one sample from the DriveLM-annotated training split: the six surround-view camera images (top) and the corresponding bird’s-eye-view (BEV) scene with ground-truth map elements and agents (ego in red, bottom). (**b**) The four-stage reasoning chain. For each stage we show the stage prompt (Q) and the DriveLM ground-truth answer used as the training target; during training, the hidden state h_k_ at the answer-end position of each stage is extracted. (**c**) Each h_k_ is injected as a gate-free residual into the corresponding component: h_1_ into the agent (detection) queries, h_2_ into the motion queries, and h_3_ (risk) and h_4_ (planning) into the ego query that is fed to the planner; the ego query thus receives both the risk and planning states. The displayed answers are supervision targets (DriveLM annotations), not model outputs, and the prompts and targets shown are representative instances (during training they are selected from the DriveLM QA by keyword matching). At inference, the language model and stage-wise injections are not executed; the GenAD decoder together with the retained BEV token path (Q-Former, SLG, and Fusion) remains active, while the hidden states are shown only schematically. Arrows in (b) denote the sequence of reasoning stages, while arrows in (c) show the flow of stage-specific hidden-state residuals into the corresponding task queries.

The four stages of queries and answers are serialized into a single sequence, and the BEV tokens V are prepended as a prefix to the input of Qwen2.5-1.5B. Figure 2 illustrates the chain and the resulting injections for one training sample. Appendix A (Table A1) lists four complete four-stage examples, including one incorrect and one ambiguous keyword-routing case.

input = [V; Stage_1_; …; Stage_4_]. (2)

Since Qwen uses decoder-only causal attention, the BEV token hidden states at the front of the sequence (positions 0–31) cannot attend to the subsequent text. Therefore, the hidden states that carry the language-reasoning content are those at the end position of each stage’s answer (h_s_ below), and these constitute the supervisory signal of our method. A causal language-modeling loss ℒ_LM_ is computed only over the answer tokens (BEV tokens and query tokens are excluded):

ℒ_LM_ = −Σ_t∈A_ log P_θ_ (x_t_|x_<t_, V). (3)

where A denotes the set of answer-token positions. Through this objective, the LLM learns the decomposed driving reasoning.

### 3.4. Stage-Wise Hidden-State Injection

At the last-token position of each stage s’s answer, we extract the LLM final-layer hidden state h_s_ ∈ ℝ ^d^_LLM_, and after a linear projection, inject it as a residual into the queries of the corresponding task decoder. The injection is a gate-free direct addition (q ← q + W_s_ h_s_), where h_s_ ∈ ℝ ^d^_LLM_ and the stage-specific projection W_s_ maps ℝ ^d^_LLM_ to the query dimension of the target decoder (256 in our setting), so that W_s_ h_s_ is dimensionally compatible with q.

Writing H for the final-layer hidden states of the language model and π_s_ for the position of the last answer token of stage s, the extracted state is given by (4) and the stage-wise injection by (5); h_1_ is broadcast to all detection queries i, h_2_ to all motion queries j, and the risk and planning states are both added to the single ego query, matching Figure 2c.

h_s_ = H[π_s_] ∈ ℝ^d^_LLM_, s ∈ {1, 2, 3, 4}, (4)

q′_agent_^(i)^ = q_agent_^(i)^ + W_1_ h_1_, q′_motion_^(j)^ = q_motion_^(j)^ + W_2_ h_2_, q′_ego_ = q_ego_ + W_3_ h_3_ + W_4_ h_4_. (5)

Concretely, the perception-stage hidden state h_1_ is added to the decoded object queries of the Agent Decoder; the motion-stage hidden state h_2_ is added to the motion queries, which are not independent embeddings but are derived from the Agent Decoder output; and the planning-stage hidden state h_4_ is added to the learned ego query, which is subsequently fed to the Motion Decoder together with the agent queries. This aligns the detection → motion → planning task flow with the corresponding language-reasoning stages. The risk-stage hidden state is injected into the same ego query, providing an additional safety-oriented context for planning.

The broadcast of a stage-level hidden state is intentional: h_1_ and h_2_ provide shared scene-level task context, while instance-specific information remains in each learned query and its attention to BEV features and preceding decoder outputs. Thus, the language residual does not replace object-to-query correspondence; it conditions the query set on the current reasoning stage while the query-specific attention mechanism continues to distinguish individual agents.

**Auxiliary feature distillation.** In addition to the stage-wise injection, which is the core mechanism of TaLS, we adopt an auxiliary feature-distillation loss that aligns the detection queries with the BEV token features as processed by the language model, in the spirit of multimodal distillation for driving [8]. The teacher is the set of LLM hidden states at the BEV token positions, V_LLM_ ∈ ℝ^32×1536^, taken with a stop-gradient; the student is the final-layer query set of the Agent (detection) decoder, Q_det_ ∈ ℝ^N×256^ with N = 300. Both are projected into a shared 128-dimensional space by small MLPs with layer normalization, and a cross-attention layer (the 32 teacher tokens as queries, the N detection queries as keys and values) produces one student representation per teacher token. Writing the ℓ_2_-normalized teacher and student representations as z^L^_i_ and z^V^_i_ over the M = N_b_ × N_q_ = 32 N_b_ pairs in a batch, where N_b_ is the batch size, the loss is the InfoNCE objective (6):

ℒ_dist_ = −(1/M) Σ_i_ log [exp(z^L^_i_ · z^V^_i_/τ)/Σ_j_ exp(z^L^_i_ · z^V^_j_/τ)], τ = 0.2, (6)

in which matching token indices form the positive pairs and all other pairs in the batch are negatives. Because the BEV token positions precede the text under causal attention (Section 3.3), this term aligns visual-token features rather than transferring language reasoning; the latter is carried by the stage-wise injection.

The overall training objective is given by (7):

ℒ = ℒ_drive_ + λ_LM_ ℒ_LM_ + λ_dist_ ℒ_dist_, ℒ_drive_ = ℒ_det_ + ℒ_map_ + ℒ_mot_ + ℒ_plan_, (7)

where the individual weights within ℒ_drive_ follow GenAD [3], λ_dist_ = 0.5, and λ_LM_ = 0.3. Which terms are active in each phase is specified in Section 3.6.

**Controlled component ablations.** To isolate the stage-wise injection from the auxiliary distillation objective, we train two variants from the same Phase-1 checkpoint. The w/o injection variant disables the four stage-wise injections while retaining the LLM forward pass, the language-modeling objective, and the distillation objective; the w/o distillation variant sets the distillation weight to zero while retaining the stage-wise injection and language-modeling objective. The variants use the same backbone, data, initialization, seed, training schedule, evaluation protocol, and all non-ablated components. Neither injection nor distillation is active in Phase 1, so all three models share the same language/Q-Former pretraining state before the controlled components are enabled.

**Stage-mismatch alignment controls.** To test whether the stage-to-task correspondence contributes beyond the presence of language-derived residuals, we evaluate two fixed one-to-one non-aligned permutations while retaining the same four hidden-state sources, destination projection modules, QA supervision, language-modeling and distillation objectives, data, seed, and training schedule. In Mismatch A, the Motion hidden state is routed to the Agent decoder, the Object/Scene hidden state to the Motion decoder, the Planning hidden state to the ego-risk injection, and the Risk hidden state to the ego-planning injection. In Mismatch B, the Planning hidden state is routed to the Agent decoder, the Risk hidden state to the Motion decoder, the Motion hidden state to the ego-risk injection, and the Object/Scene hidden state to the ego-planning injection. Each permutation is fixed for the entire run; only the source-to-destination routing of the residual-injection pathway is changed, while the original language-modeling and distillation targets remain unchanged. The aligned reference is taken from the original TaLS experiment reported in Section 4.1, and the mismatch variants follow the same model architecture, dataset split, three-phase training schedule, and evaluation protocol.

### 3.5. Train/Inference Asymmetry and Mechanism Analysis

**Training.** On QA-annotated frames, the TD-QA chain provides the stage hidden states used by the injection pathway. The active objectives are phase-dependent as detailed in Section 3.6: Phase 1 uses the language-modeling objective on QA frames and Q-Former reconstruction on non-QA frames; Phase 2 optimizes the driving losses and the auxiliary distillation objective with the LLM frozen; and Phase 3 jointly optimizes the driving losses, the language-modeling objective on QA frames, and the distillation objective. Since the BEV encoder and image backbone are frozen throughout, the trainable components retained at inference are the task decoders and heads together with the BEV token modules. Because each per-stage hidden state is added to the decoder queries while the driving loss is minimized, gradients from the driving objectives update the decoder weights under the injected context during Phases 2 and 3.

**Inference.** Without QA annotations, the autoregressive LLM (Qwen2.5-1.5B) is not executed, and the stage-wise hidden-state injection is omitted (LLM-free); however, the BEV Q-Former and the two BEV token augmentation modules (SLG and Fusion) are retained in the inference graph. The deployment path is therefore the GenAD E2E decoder plus the retained BEV token path (Q-Former, SLG, Fusion), while the language-generation/injection branches and other training-only loss modules, including the Q-Former reconstruction head and the internal distillation projections, are absent from or inactive in deployment. The language branch acts as a training-time representational supervisor rather than an inference-time generator: under teacher forcing, it shapes the stage representations used during training, and free-form language generation is never used at inference. This is consistent with the training-only design. The controlled w/o injection experiment in Section 4.4 provides a direct test of this train/inference asymmetry: both models omit language residuals at inference, while only the full model receives stage-wise injection during training.

**Mechanism analysis (measurements on the trained model).**

Although the BEV token residual has a small relative magnitude (r_SLG_ = 1.9%, r_Fusion_ = 0.5%), disabling the path increases L2 from 0.517 to 0.544, confirming its functional importance for trajectory accuracy. In contrast, the collision rate remains below the GenAD baseline after pruning, indicating that part of the collision improvement persists independently of the retained BEV token path.

The two retained modules are defined in (8), where Φ_SLG_ back-projects the BEV tokens V onto the BEV grid using the Q-Former cross-attention weights, CA denotes cross-attention with the detection queries as queries and V as keys and values, and g_S_, g_F_ are learned scalar gates. The prune ablation of Section 4.4 sets g_S_ = g_F_ = 0 at inference. The relative contribution of each module, defined in (9) where Δ denotes each module’s residual, is r_SLG_ = 1.9% and r_Fusion_ = 0.5%.

B′ = B + g_S_ Φ_SLG_(V), Q′_agent_ = Q_agent_ + g_F_ CA(Q_agent_, V). (8)

r_SLG_ = ‖g_S_ Δ_SLG_‖/‖B‖, r_Fusion_ = ‖g_F_ Δ_Fusion_‖/‖Q_agent_‖. (9)

### 3.6. Training Strategy

We adopt three-phase training. (1) **Phase 1** (5 epochs, base learning rate 1.2 × 10^−3^): the task decoders are frozen while the Q-Former and LoRA adapters are optimized. QA-annotated frames use the token-level language-modeling loss ℒ_LM_; for non-QA frames, the Q-Former instead receives a reconstruction objective, MSE(qformer_recon_head(V), average-pooled BEV) with weight 0.1, so that visual-token learning continues without language injection. (2) **Phase 2** (5 epochs, 1.4 × 10^−4^): the task decoders, heads, SLG, Fusion, and stage-wise injection projections are trained with the driving losses and ℒ_dist_ while the language model is frozen. (3) **Phase 3** (10 epochs, 3 × 10^−5^): all trainable components are jointly fine-tuned with the driving losses, ℒ_LM_ (weight 0.3), and ℒ_dist_. On non-QA frames, stage-wise injection is inactive, and ℒ_LM_ is exactly zero in Phases 2–3.

This mixed supervision uses all 28,130 training frames: the 4072 QA-annotated frames contribute the stage-wise language signal, whereas the remaining frames continue to train the vision-driving path (and the Phase-1 Q-Former reconstruction objective). This design preserves the full driving dataset while restricting language-dependent computation to frames for which DriveLM supervision is available.

### 3.7. Experimental Setup

**Dataset and metrics.** We evaluate on the nuScenes [23] validation set. Planning is reported with L2 error and collision rate (box collision) under two aggregation protocols: TemAvg (cumulative average over all waypoints within the 0–N s interval), the protocol used by our baseline GenAD (following VAD), and NoAvg (the value at the N-s time point alone). We take TemAvg as the primary metric since it matches our baseline, and report NoAvg alongside it. In both cases, the reported “Avg.” is the arithmetic mean of the 1 s, 2 s, and 3 s values, computed before rounding. The main benchmark results are reported to two decimal places for consistency with prior work, whereas the later ablation tables use three decimal places to preserve smaller within-model differences; the reproducibility and stage-mismatch analyses retain the precision needed for mean ± SD reporting and close controlled comparisons. Relative improvements quoted in the text are computed from the unrounded values. Because the per-timestep aggregation is sometimes labeled “ST-P3” [14] in code and its exact definition varies across prior works, we standardize on the explicit terms TemAvg/NoAvg to avoid ambiguity. The benchmark values under these two protocols are presented in Table 3 and Table 4.

Planned trajectories consist of waypoints at 0.5 s intervals, so an N-second horizon contains 2N waypoints; the collision rate is computed by placing the ego box (1.85 m × 4.084 m) at each evaluated waypoint and testing intersection with the ground-truth occupancy of the surrounding agents at the corresponding time step.

**Implementation.** Backbone ResNet-50 [21], BEV 100 × 100, LLM Qwen2.5-1.5B-Instruct [24] (LoRA [25] *r* = 16, *α* = 32, target modules: q_proj and v_proj), 32 BEV Q-Former queries. The implementation was based on Python 3.10 and PyTorch 2.0.1.

**Experimental conditions.** The reference training runs underlying the main benchmark and ablation experiments (Section 4.1, Section 4.2, Section 4.3 and Section 4.4) were performed on a single NVIDIA A100-SXM4-80GB GPU (NVIDIA Corporation, Santa Clara, CA, USA). The three-phase curriculum used a per-GPU batch size of 16 in Phase 1 and 8 in Phases 2–3 without gradient accumulation, so the effective batch size equals the per-GPU value. Input images (1600 × 900) were scaled by 0.4 and padded to a multiple of 32, giving a 640 × 384 input. Optimization used AdamW (weight decay 0.01) with the per-phase learning rates detailed in Section 3.6, and the curriculum ran for 5 + 5 + 10 = 20 epochs. Inference efficiency was measured on a single A100-SXM4-80GB at batch size 1 in FP32 using the same six-camera 384 × 640 tensor input; latency uses CUDA events and excludes data loading, host-to-device transfer, metric computation, and result serialization. The robustness study in Section 4.5 uses the same frozen checkpoints and metric pipeline at batch size 1. Following the fair-comparison setting of VAD [2] and GenAD [3], ego-status inputs were not provided to the planner in either the GenAD baseline or any TaLS variant; only the standard navigation command was used. Unless otherwise stated, the main benchmark, controlled ablations, and robustness analyses use the same reference training setup so that each comparison isolates the intended factor; independent three-seed repetitions are reported separately in Section 4.6.

**Multi-seed protocol.** To assess training variability, we evaluate both GenAD and TaLS using the same three random seeds (0, 1, and 2) and report the arithmetic mean ± sample standard deviation (ddof = 1). The architecture, training recipe, dataset split, ego-status setting, evaluation pipeline, and final-checkpoint selection rule are kept fixed within each method across all three seeds. The main per-horizon benchmark is reported separately from these three-seed statistics, which serve as a complementary reproducibility analysis.

**Routing-analysis protocol.** To characterize the stage assignment used during training, we re-executed the exact routing function over all 4072 annotated frames (254,522 DriveLM QA pairs) and applied the same fixed form-level semantic rubric to every keyword-gated (QA, stage) candidate assignment. Because a QA can satisfy more than one stage keyword set, this produced 263,716 candidate-stage assignments. The rubric classifies an assignment as clear when the routed stage matches the primary semantic target, ambiguous when the QA is semantically compatible with more than one neighboring stage, and incorrect when the primary target belongs to another stage. To reflect the router’s training-time operation, which selects one candidate for each stage in every annotated frame, we report a frame-weighted expected distribution over the eligible candidates, yielding 4072 frame-level contributions per stage (16,288 in total) without QA subsampling. To account for the stochastic candidate selection used by the router, we repeated the routing selection over all 4072 annotated frames using 20 routing RNG seeds (0–19) and measured how often two stages selected the same QA content within a frame.

**Model complexity.** We distinguish the full training checkpoint, the retained deployment graph, and the subset of parameters executed during an LLM-free forward pass. Under a unified parameter-element count (buffers excluded), GenAD contains 47.2439 M retained deployment parameters and 41.9995 M active inference parameters. TaLS contains 1778.7998 M parameters in the training checkpoint, 230.9376 M after removal of training-only modules, and 143.8914 M active inference parameters under forward-hook tracing. The training-only Qwen2.5-1.5B/LoRA branch and stage-injection modules are not executed at inference. The retained inference overhead is characterized directly in Section 4.3 using estimated supported-operator FLOPs, CUDA-event latency, and peak allocated GPU memory.

## 4. Results

### 4.1. Planning

**Figure 3:**
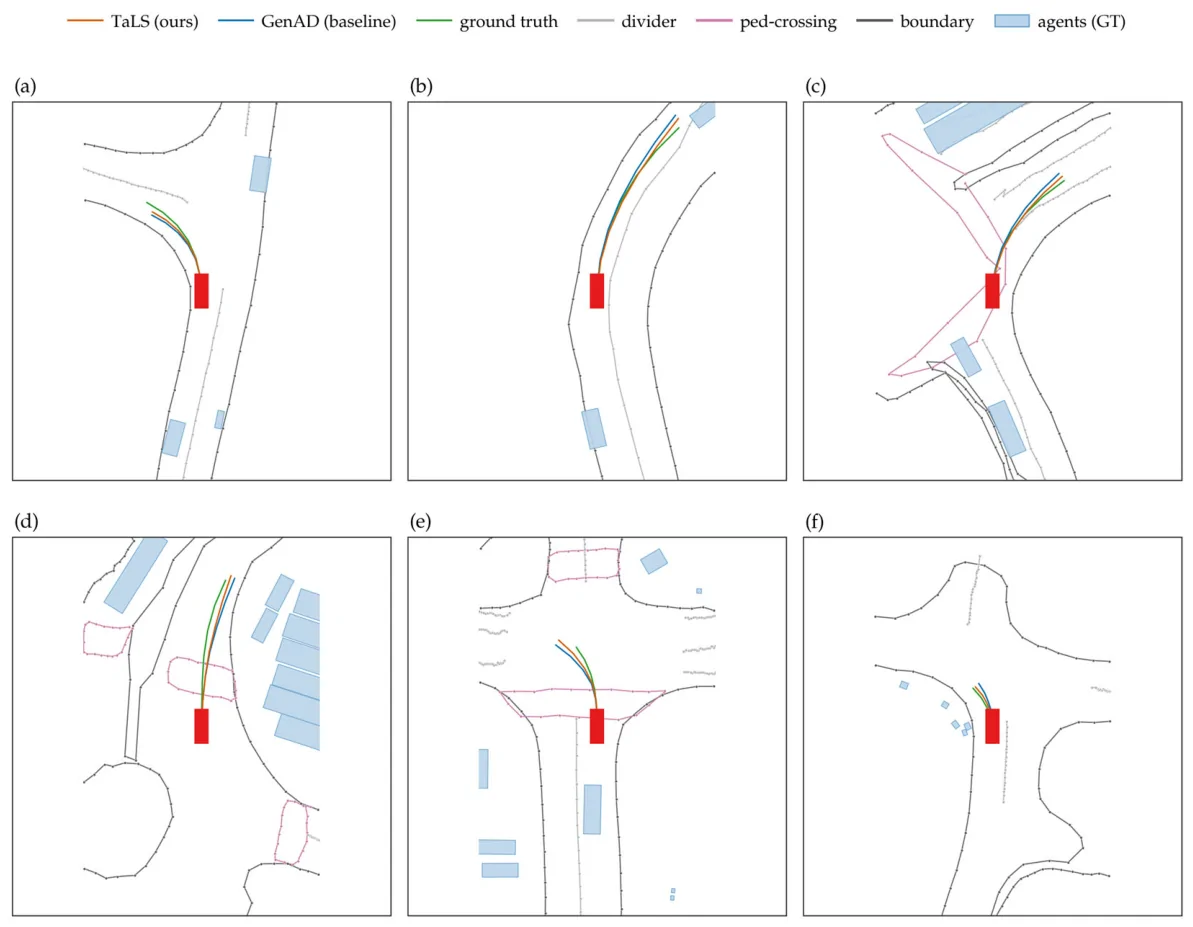
Qualitative comparison of planned 3 s ego trajectories on the nuScenes validation set. Each panel is a bird’s-eye view centered on the ego vehicle (red box), covering ±15 m laterally and ±24 m longitudinally. Light-blue boxes are ground-truth agents, and the vectorized map elements are the model’s own predictions, with lane dividers in light gray, pedestrian crossings in pink, and road boundaries in dark gray (polyline vertices shown as dots). Three 3 s trajectories are overlaid: TaLS (orange) and the GenAD baseline (blue), each planned for the selected driving command, together with the ground-truth trajectory (green). Six representative scenes are shown: (**a**) left turn, (**b**) right turn, (**c**) right turn, (**d**) right curve, (**e**) left turn, (**f**) left turn. The examples span turns and curved road segments, including intersections with pedestrian crossings and surrounding agents, and across these scenes TaLS produces smooth, map-consistent trajectories that follow the ground-truth path closely.

Table 3 and Table 4 report the main open-loop planning results under the two aggregation protocols. Compared with the GenAD baseline, TaLS reduces the open-loop box-collision metric under both protocols (TemAvg 0.19 → 0.13, −31.3% relative; NoAvg 0.32 → 0.27) while maintaining comparable trajectory accuracy (TemAvg L2: 0.52 for both models; NoAvg L2: 0.96 → 0.95). TaLS also achieves the lowest quoted collision rate at the 3 s horizon and on average within each protocol block. Because the results of prior methods were obtained in their respective experimental environments, these cross-paper values are provided as contextual references, whereas the GenAD–TaLS comparison constitutes the controlled evaluation in this study. A complementary three-seed analysis is reported in Section 4.6 to assess reproducibility across training initializations. Representative planned trajectories are shown in Figure 3.

### 4.2. Perception

Table 5 summarizes the detection and map-perception results. TaLS maintains perception performance comparable to the GenAD baseline, indicating that the observed planning differences are not accompanied by a material change in the underlying perception metrics.

### 4.3. Efficiency

At inference, the autoregressive LLM and stage-wise injection projections are removed completely. Forward tracing identifies 143.9 M active parameters for TaLS versus 42.0 M for GenAD; the larger count reflects the retained Q-Former together with SLG and Fusion. Under the matched A100-SXM4-80GB, batch-1, FP32 protocol in Table 6, TaLS runs at 15.6 FPS compared with 17.4 FPS for GenAD, corresponding to a 9.9% throughput reduction. The prune analysis in Section 4.4 shows that this retained path contributes directly to trajectory accuracy, so its inference cost corresponds to a functional component rather than unused language-model computation.

The measured deployment cost is modest in computation but larger in resident parameters: TaLS adds 7.9 GFLOPs (+4.0%) and approximately 6.3 ms (+10.9%) of mean forward latency, while active parameters increase from 42.0 M to 143.9 M and peak allocated GPU memory from 0.52 to 1.22 GiB. The latency measurement uses 100 warmup samples followed by 1000 timed samples in each of three rounds with CUDA events and identical sample IDs; host-to-device transfer and data loading are excluded. The increase in peak memory is dominated by the larger resident parameter footprint, while the measured dynamic-memory increment remains comparable between the two models.

Forward-hook tracing confirms that Qwen/LoRA, the stage-wise language-injection projections, and the Q-Former reconstruction head are not executed during evaluation, whereas the Q-Former, spatial grounding, and query-fusion modules remain active. On the controlled validation sample used for the stripping-equivalence test, removing the training-only modules yields bit-exact outputs (maximum absolute difference 0 across the compared output tensors), confirming that this evaluated deployment path does not depend on autoregressive LLM inference. FLOPs are reported as supported-operator estimates because fvcore does not count some custom CUDA and element-wise operations; the dominant unsupported deformable-attention operations are shared by both models, while small unsupported operations may also occur in TaLS-specific modules.

These measurements characterize the model-side inference cost under a controlled deployment-oriented setting. By isolating the network forward pass from data loading, host-to-device transfer, metric computation, and result serialization, the protocol enables a direct and reproducible comparison of the inference overhead introduced by TaLS. Under this setting, TaLS operates at 15.6 FPS while avoiding autoregressive LLM inference at deployment.

**Deployment perspective.** The measured inference performance demonstrates that TaLS maintains 15.6 FPS on the tested server-class GPU without requiring autoregressive LLM generation. Its larger active parameter footprint motivates further optimization for resource-constrained automotive hardware. DIR-BHRNet [26] illustrates how lightweight convolutional building blocks and balanced network structures can enable real-time multiperson pose estimation on smartphones. Although addressing a different vision task, this work provides a relevant example of resource-efficient visual network design. Accordingly, Q-Former slimming, pruning, quantization, and hardware-aware acceleration offer promising directions for deploying the retained BEV token modules on lower-compute automotive platforms.

### 4.4. Ablation (Planning)

**BEV token path prune (no retraining).** On the trained model, we fixed the SLG and Fusion gates to 0 (output-equivalent to removing the modules) and evaluated at inference. Table 7 reports the resulting inference-time pruning comparison.

**Figure 4:**
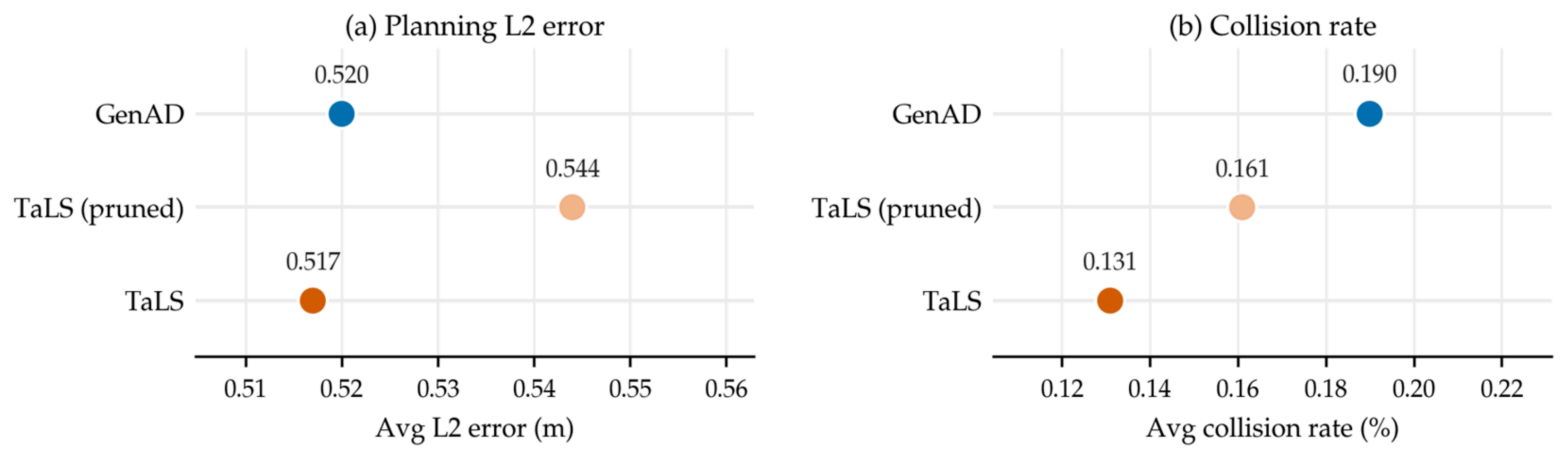
Prune ablation of the retained BEV token path (SLG and Fusion) on the nuScenes validation set (TemAvg protocol; inference-time gate zeroing, no retraining). Each panel compares the GenAD baseline, TaLS with the SLG and Fusion gates set to zero at inference (Pruned), and the full TaLS model. (**a**) Zeroing the path raises the planning L2 error above the baseline (0.520 → 0.544 m for Pruned, versus 0.517 m for the full model), showing that the path is load-bearing for trajectory accuracy even though its learned gates are near zero. (**b**) The collision rate remains clearly below the baseline after pruning: the pruned model reduces it by 15.7% relative to GenAD (0.190 → 0.161), compared with 31.3% for the full model (0.190 → 0.131), and it stays below the baseline at every horizon (0.107/0.147/0.228 versus 0.137/0.181/0.254). The contrast between the two panels highlights the different roles of the retained BEV token path: disabling the path substantially affects L2, confirming its importance for trajectory accuracy, whereas a considerable portion of the collision-rate improvement persists after pruning.

Disabling the inference-time BEV token path (SLG and Fusion, gate → 0) increases L2 from 0.517 to 0.544, with the same trend observed across all prediction horizons, confirming the functional importance of this path for trajectory accuracy despite its small residual magnitude (gate ≈ 0.01). The relative contributions of the two modules are r_SLG_ = 1.9% and r_Fusion_ = 0.5%. In contrast, the collision rate remains below the GenAD baseline after pruning: it is reduced by 15.7% relative to GenAD (0.190 → 0.161), compared with 31.3% for the full model (0.190 → 0.131), and remains lower at every horizon (0.107/0.147/0.228 versus 0.137/0.181/0.254). These results show that the retained BEV token path is particularly important for L2, whereas part of the collision improvement persists after the path is disabled, consistent with partial encoding in the retained decoder weights. Figure 4 visualizes this ablation.

**Language supervision versus BEV token modules.** To test whether the planning gains stem from the language supervision itself or merely from the additional BEV token modules, we trained a variant of TaLS without language supervision, denoted TaLS w/o language. It is identical to TaLS in architecture, training schedule, data, initialization, and budget, except that the language branch is disabled: the autoregressive LLM forward pass, the LoRA adapters, the language-modeling loss, the auxiliary feature-distillation loss, and the stage-wise hidden-state injection are all removed (equivalently, every training sample is treated as having no QA), while the BEV Q-Former, SLG, and Fusion modules are retained. In the full model, Phase 1 uses the language-modeling objective on QA-annotated frames and the Q-Former reconstruction objective on non-QA frames. In the w/o language variant, all frames follow the non-QA path, so Phase 1 updates the Q-Former with the same reconstruction objective (weight 0.1) while the other components remain frozen. The Q-Former is therefore carried into Phase 2 without BEV–language alignment, which follows directly from removing language supervision rather than from an incidental difference in setup. The phase lengths, optimizer settings, and learning rates in Phases 2 and 3 follow the full-model schedule, while all language-dependent modules and losses remain disabled. This broad ablation removes stage-wise injection and auxiliary distillation jointly; their individual effects are therefore separated later by the controlled variants reported below. Table 8 compares GenAD, TaLS w/o language, w/o decomposition, and TaLS under this common setting.

In the controlled TemAvg ablation, the collision rate changes from 0.190 for GenAD to 0.175 with the BEV token modules and to 0.131 with the full TaLS model. This comparison associates the language-supervised components with an additional reduction in the collision metric, while the L2 change is already present with the BEV token modules (0.520 → 0.517) and is preserved in the full model. Because this ablation jointly removes several language-dependent components, it is interpreted as evidence of complementary roles rather than as an isolated causal estimate of language semantics.

**Decomposed versus undecomposed language supervision.** The preceding controlled ablation associates the language-dependent training components with a lower collision metric, but it does not determine whether the difference follows from the task decomposition or simply from exposing the model to driving-related language during training. We therefore train a non-decomposed variant, denoted TaLS w/o decomposition, that is identical to the full model in every other respect—BEV token modules, LLM and LoRA adapters, language-modeling and distillation losses, number of injection sites, training budget, data, and schedule—but removes the four-stage structure: the four DriveLM answers are concatenated into a single generic question–answer pair without stage markers, and the resulting single answer-end hidden state is broadcast to all query types instead of four stage-aligned states being injected into their corresponding queries. The language content and token budget are thus held fixed, and only the structure is removed.

Within this TemAvg ablation ladder, the collision rate decreases from 0.190 for GenAD to 0.175 with the BEV token modules, 0.144 with non-decomposed language supervision, and 0.131 with the full TaLS model. The observed 0.144 → 0.131 difference is consistent with an additional contribution from stage-aligned decomposition within this mechanism-focused comparison. The decomposition also provides explicit stage-to-module traceability through its query-aligned injection design. The magnitude of these ablation differences is therefore interpreted as an observed controlled trend rather than as a standalone statistical estimate.

**Three-phase curriculum versus single-stage training.** The full model is trained with a three-phase curriculum: the LoRA adapters and BEV Q-Former are trained first, then the decoders together with the SLG, Fusion, and injection modules while the LLM is frozen, and finally all trainable components are fine-tuned jointly. To test whether this schedule is necessary, we train a single-stage variant that is architecturally identical to the full model—the same four-stage decomposition and aligned injection—but jointly optimizes all trainable components from the pretrained GenAD initialization for the same total budget of 20 epochs. To keep the comparison fair, the single-stage schedule uses fixed module-wise learning rates chosen to match the nominal learning-rate scales of the curriculum: a base rate of 1 × 10^−4^ for the pretrained decoders and heads, with the BEV Q-Former at 1.2 × 10^−3^, the LoRA adapters at 3.6 × 10^−4^, and the SLG, Fusion, and injection projections at 1.5 × 10^−4^ (effective batch size 8); the BEV encoder and image backbone remain frozen as in the main model. Table 9 summarizes the corresponding training-schedule results.

Table 9 demonstrates that the main benefit of the three-phase curriculum appears in the collision metric: the average collision rate decreases from 0.338 for single-stage joint training to 0.131 for the curriculum, while L2 remains comparable (0.508 versus 0.517 m). Training diagnostics further show that the single-stage run does not exhibit numerical divergence: its total loss decreases from 6.63 to 5.56 over training and, after the initial learning-rate warmup transient, the logged gradient norm remains bounded. The performance gap is therefore better interpreted as sensitivity to the optimization schedule than as optimization failure. Because the set of trainable parameters differs across curriculum phases, gradient-norm magnitudes are not used as a direct measure of relative stability between the two schedules.

**Figure 5:**
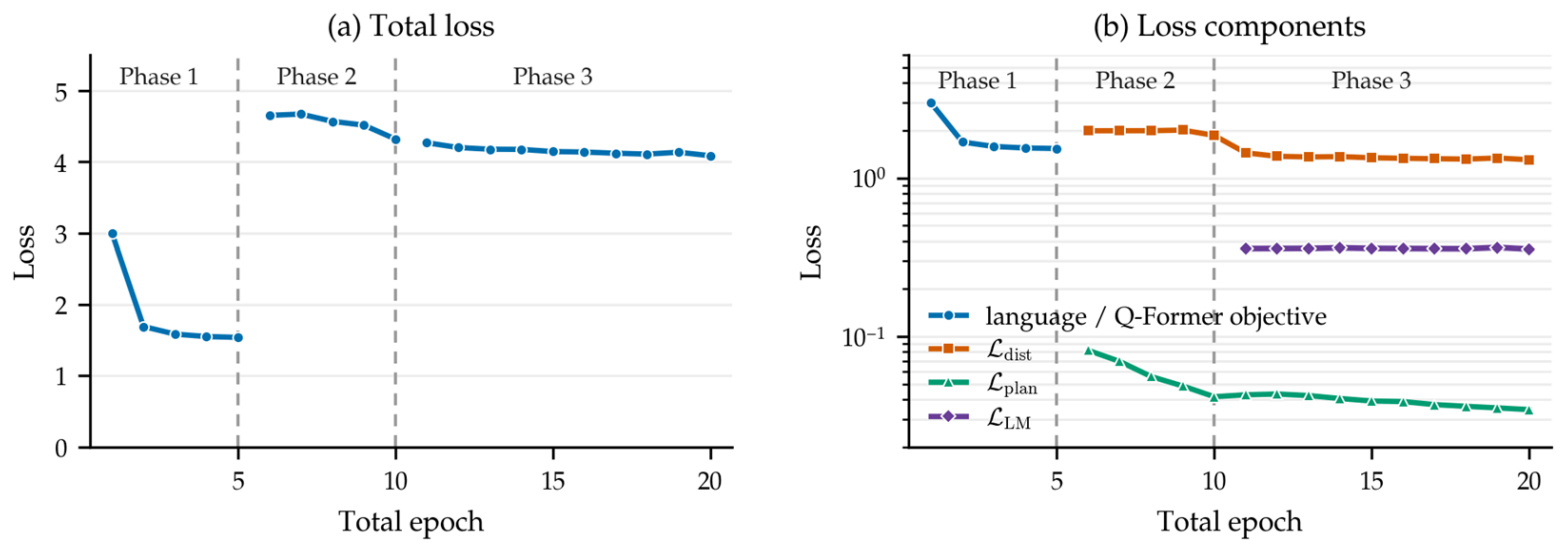
Training dynamics of the three-phase curriculum for the reference training run. (**a**) Total loss is shown separately within each phase because the active losses and trainable modules change at the phase boundaries. (**b**) Active loss components on a logarithmic *y*-axis. The Phase-1 language-side log also contains the Q-Former reconstruction objective used for non-QA samples (85.5% of training frames), so it is labeled as the language/Q-Former objective rather than as a pure language-modeling loss. Inactive objectives are omitted rather than plotted as zeros.

Figure 5 reports the three-phase training curves reconstructed from the reference training log. The monitored objectives remain non-divergent and show an overall decreasing trend within each active phase. The apparent jump in total loss at phase boundaries is expected because both the active objectives and the trainable module set change with the curriculum; total-loss magnitudes are therefore interpreted within, rather than across, phases.

**Figure 6:**
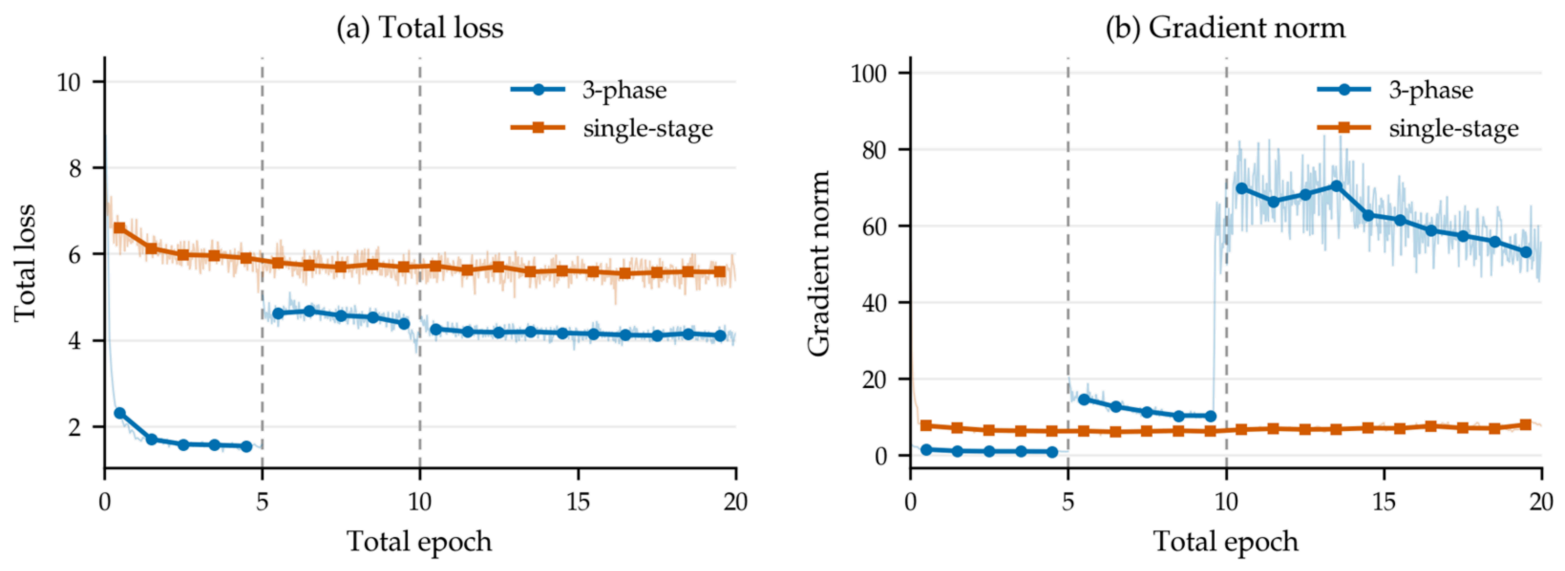
Optimization diagnostics for three-phase and single-stage training in the reference comparison. (**a**) Total loss and (**b**) logged gradient norm. Absolute total-loss and gradient-norm magnitudes are not interpreted as direct cross-schedule stability measures because the active objectives and trainable parameter sets differ; the curves are used to verify non-divergent optimization and to contextualize the collision-rate gap in Table 9.

Figure 6 directly contrasts the no-curriculum single-stage run with the three-phase schedule. The single-stage loss decreases throughout the 20-epoch run, and its post-warmup gradient norm remains bounded, confirming that the lower collision performance in Table 9 is not caused by numerical divergence. The phase separators apply only to the three-phase run; the single-stage variant optimizes all trainable components continuously for the full 20 epochs.

**Stage-wise injection versus auxiliary distillation.** To separate the effect of the injected language states from the auxiliary distillation objective, we compare the full TaLS model with two controlled variants. The w/o injection model keeps the LLM forward pass, language-modeling objective, and distillation objective but disables all four stage-wise injections; the w/o distillation model retains the injections and language-modeling objective while setting the distillation weight to zero. Training logs verify the intended controls: the distillation loss is exactly zero throughout the logged Phase 2/3 for w/o distillation, while the language-side objective remains essentially unchanged across the three runs. Table 10 reports the resulting component ablations.

Removing stage-wise injection leaves trajectory L2 comparable (0.512 versus 0.517 m) but increases the TemAvg collision rate from 0.131% to 0.205%, exceeding the GenAD baseline value of 0.190%. Because the language-modeling and distillation objectives remain active in this variant, this controlled comparison identifies stage-wise injection as a major contributor to the observed collision-rate reduction. In contrast, removing the auxiliary distillation term changes L2 by only +1.2 mm and does not degrade the collision metric in the tested comparison (0.117% versus 0.131%). The secondary NoAvg protocol shows the same direction for injection removal (0.274% to 0.345%), while detection performance remains essentially unchanged across the compared variants. These mechanism-focused ablations are interpreted as controlled trends rather than as standalone statistical performance estimates.

**Figure 7:**
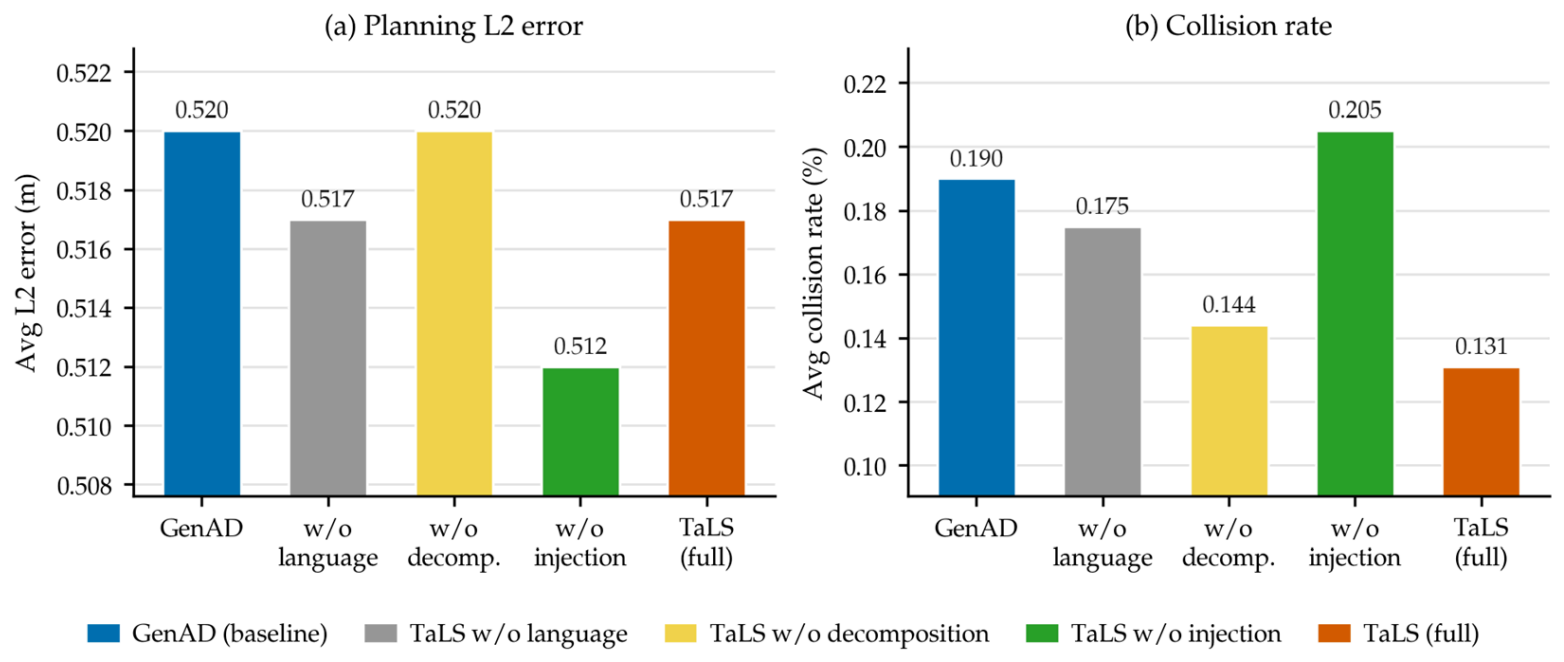
Summary of the main ablation variants on the nuScenes validation set under the primary TemAvg protocol (reference run); lower is better in both panels. (**a**) Planning L2 error and (**b**) collision rate. Both y-axes focus on the observed value ranges to make the small between-variant differences legible, and the exact values are printed above each bar. The figure consolidates two complementary controlled comparisons from Section 4.4: Table 8 evaluates language removal and task decomposition, while Table 10 isolates stage-wise injection using the shared Phase-1 checkpoint. Together, the two panels provide a compact overview of how the proposed language-supervision components affect trajectory L2 and the collision metric.

**Summary of the principal ablations.** Figure 7 brings together the five variants most directly associated with the proposed mechanism. Among the five variants summarized in Figure 7, full TaLS achieves the lowest collision metric (0.131%), while removing stage-wise injection increases it to 0.205%, above the 0.190% GenAD reference value. The L2 values remain closely grouped (0.512–0.520 m), highlighting that the clearest separation among these variants appears in the collision metric while trajectory accuracy remains comparable.

### 4.5. Robustness Under Visual Degradation

To assess whether the planning improvement persists when the camera input is degraded, we perform a zero-shot robustness evaluation with frozen checkpoints and no corruption-aware retraining. Four deterministic pixel-domain degradations are applied before normalization using one fixed setting per degradation type: Gaussian noise (σ = 25 on the 0–255 scale), low-light intensity scaling (0.35), motion blur (kernel size 15), and CAM_FRONT dropout, implemented by replacing only the front view with the normalization mean so that the normalized input carries no image information. Both TaLS and GenAD are evaluated on the full nuScenes validation split using the same TemAvg/NoAvg planning metrics as the main experiments. Table 11 summarizes the results under the primary TemAvg protocol.

TaLS retains a lower absolute collision rate than GenAD in the clean condition and under all four tested degradations. The separation is largest under motion blur (0.177% versus 0.382%) and remains substantial under Gaussian noise (0.163% versus 0.283%); TaLS also remains lower under low light (0.152% versus 0.199%) and CAM_FRONT dropout (0.199% versus 0.239%). For Gaussian noise, low light, and motion blur, L2 changes by at most 0.013 m relative to each model’s clean result, whereas removing the front view increases L2 for both models by approximately 0.19 m. The secondary NoAvg protocol shows the same ordering in absolute collision rate across all five conditions. These fixed-setting results are interpreted as observed robustness trends rather than as statistical superiority claims.

### 4.6. Reproducibility, TD-QA Routing, and Stage-Alignment Control

**Multi-seed reproducibility.** We repeated both GenAD and TaLS with seeds 0, 1, and 2, using each method’s fixed training recipe with the same random-seed values and a common evaluation protocol. The per-seed comparison shows heterogeneous behavior across initializations rather than a uniform shift: TaLS lowers the TemAvg collision rate in seeds 0 and 2 (0.1905 → 0.1308% and 0.2301 → 0.2138%), whereas seed 1 shows the opposite direction (0.2947 → 0.3880%). The higher seed-1 value materially increases both the mean and variance, yielding a three-seed aggregate of 0.238 ± 0.053% for GenAD and 0.244 ± 0.131% for TaLS. The seed-1 TaLS training log nevertheless shows non-divergent optimization and final training objectives comparable to seed 2, which is consistent with sensitivity to training initialization rather than numerical optimization failure. The same pattern is visible in trajectory error: seeds 0 and 2 remain close to their corresponding GenAD values. Under the secondary NoAvg protocol, TaLS yields a lower or equal NoAvg collision rate across all three seed settings (0.3191 → 0.2735%, 0.5405 → 0.5274%, and 0.4167 → 0.4167%), producing a lower three-seed mean collision rate (0.406 ± 0.127% versus 0.425 ± 0.111%). Mean perception performance also remains essentially unchanged. Taken together, the three-seed results show that collision-rate improvements are observed across multiple initializations, while their magnitude depends on the initialization and aggregation protocol. Table 12 summarizes the three-seed statistics.

**TD-QA routing characteristics.** The exhaustive census covers all 263,716 keyword-gated candidate-stage assignments generated from the 4072 annotated frames. Because the training router ultimately selects one candidate per frame for each stage, Table 13 reports the frame-weighted expected distribution, in which every annotated frame contributes equally within a stage. This view directly reflects the composition of the TD-QA supervision presented by the training procedure while retaining the full-dataset coverage of the census.

Under the frame-weighted training-time distribution, 52.8% of assignments are clear, and 32.3% are ambiguous. The ambiguous cases are dual-eligible questions whose content is consistent with two neighboring stage definitions; a representative example is “Are there moving cars to the front of the ego car?”, which jointly describes object presence (Object/Scene) and motion state (Motion). The remaining 14.9% are incorrect under the fixed rubric, meaning that another stage is judged to be the better primary match rather than that the question is unrelated to the assigned stage. These cases are highly structured: Object/Scene–Motion exchanges account for 58.3% of the incorrect assignments (8.71% of all injected questions), and both stages draw from the same DriveLM perception list and describe the same scene entities at complementary levels. Risk–Planning exchanges account for a further 40.0% (5.97% overall); because both hidden states are added to the same ego query through separate stage-specific projections (Equation (5), Figure 2c), these exchanges preserve the receiving decoder while varying the supervisory context. Only 1.6% of the incorrect assignments—0.25% of all injected questions—cross both the source group and the injection site. Thus, the census indicates that departures from the primary-stage rubric are overwhelmingly localized to semantically adjacent stage pairs. This naturally occurring local overlap is distinct from the stage-mismatch controls below, which apply fixed non-aligned permutations systematically to every stage signal throughout training.

Across 20 routing RNG draws, the same QA content was selected for two stages in an average of 7.44% of the 4072 annotated frames (5.13% for Risk–Planning and 2.44% for Object/Scene–Motion). These selections do not merge the injection paths: the corresponding states are extracted at different sequence positions and passed through stage-specific projections. The result therefore characterizes overlap in the supervision content of neighboring stages rather than a change in the four stage-specific injection destinations. Complementary controlled conditions further characterize assignment sensitivity: removing the decomposition entirely yields 0.144% collision (Table 8), while the two distinct systematic stage-mismatch permutations each round to 0.141% under the primary TemAvg protocol (Table 14), compared with 0.131% for aligned TaLS and 0.190% for GenAD. Thus, aligned task decomposition retains the lowest TemAvg collision metric among these routing-focused comparisons while trajectory accuracy remains comparable.

**Stage-mismatch alignment controls.** We next evaluate the two fixed non-aligned permutations defined in Section 3.4. These controls are qualitatively different from the localized routing overlap measured above because they systematically remap every stage signal throughout training rather than reflecting occasional ambiguity between neighboring QA definitions. Under the primary TemAvg protocol, aligned TaLS attains 0.517 m L2 and 0.131% collision, whereas Mismatch A gives 0.517 m and 0.141% and Mismatch B gives 0.514 m and 0.141%, respectively. Across the evaluated configurations, aligned TaLS achieves the lowest TemAvg collision metric, while trajectory error remains comparable across the three configurations. These observations are consistent with stage-to-task correspondence contributing to the collision-oriented benefit of TaLS under the primary evaluation protocol. Under NoAvg, Mismatch A gives 0.944 m L2 and 0.352% collision, while Mismatch B remains close to the aligned reference at 0.941 m and 0.273% (aligned: 0.947 m and 0.274%). Both permutations preserve perception quality, indicating that the TemAvg separation is not accompanied by a material perception change. Together, the two controls show that stage alignment is most clearly reflected in the paper’s primary TemAvg collision metric, while L2 and perception remain stable across the tested routings.

**Scope and limitations.** All planning results in this study are open-loop metrics computed against recorded nuScenes trajectories; they do not execute the predicted trajectory in a reactive environment and therefore do not establish closed-loop driving safety. The collision metric is sensitive to aggregation convention and initialization, as illustrated by the TemAvg/NoAvg and three-seed analyses. We consequently restrict the claims to open-loop planning behavior and leave closed-loop evaluation with reactive agents and feedback control to future work. The w/o injection comparison demonstrates that a benefit learned with training-time residuals can persist after the residuals are removed at inference, while the two stage-mismatch controls provide complementary evidence that aligned stage-to-task routing contributes to the stronger collision reduction observed under the primary TemAvg protocol. The present results support the effectiveness of the tested gate-free residual injection design, while alternative strategies such as stochastic injection dropout, gradual residual removal, or learned gating remain worthwhile directions for investigating the train/inference transition.

## 5. Conclusions

This paper presented TaLS, a training-only language-supervision framework for end-to-end autonomous driving. TaLS decomposes driving-oriented QA into perception, motion, risk, and planning stages and injects the corresponding language hidden states into the task queries during training, while removing the autoregressive LLM and stage-wise language injection at inference. In the reference run on the nuScenes open-loop benchmark, TaLS reduces the TemAvg open-loop box-collision metric by 31.3% relative to the GenAD baseline under the same experimental setting while maintaining comparable trajectory and perception performance. Controlled ablations identify stage-wise injection and task decomposition as contributors to the observed collision reduction. Across the evaluated stage-routing configurations, aligned TaLS achieves a lower TemAvg collision metric (0.131%) than two distinct fixed permutations (0.141% each), with comparable trajectory L2, providing complementary evidence for the value of stage-to-task correspondence. The prune analysis indicates complementary roles for the training-time language supervision and the retained BEV token pathway: part of the collision-rate improvement persists after pruning, whereas the retained pathway contributes more strongly to trajectory accuracy. The three-seed analysis further shows that the TemAvg effect is initialization-dependent, with improvements in two of three runs and closely matched aggregate means, while the NoAvg collision rate is lower or equal across all three runs and lower on average. The visual-degradation evaluation complements this result by showing a lower absolute collision rate under all four tested degradations. TaLS therefore demonstrates that structured language supervision can shape an E2E driving model without requiring autoregressive LLM inference at deployment. The retained inference modules introduce only a modest increase in FLOPs and inference latency relative to GenAD. Future work will extend the framework to closed-loop evaluation with reactive agents and feedback control.

## Figures and Tables

**Table 1:** Qualitative comparison with closely related language-based end-to-end driving methods. “Training-time language cost” indicates how the language model or its generated supervision participates during training; “Inference language cost” indicates whether language-model computation remains in the deployment loop.

Method	Inference Language Cost	Training-Time Language Cost	Injection/ Alignment Location	Supervision Structure
VLP [6]	None from the language branch; VLP is training-only	Frozen LM plus training-only adaptation/contrastive objectives	Agent BEV features and ego planning query are aligned with text features	Agent-wise and sample-wise vision-language contrastive alignment
VLM-AD [7]	No VLM required at inference	VLM-generated supervision plus auxiliary-head training	Auxiliary reasoning/action heads attached to the ego representation	VLM-generated free-form reasoning and structured action supervision
DiMA [8]	MLLM is optional; the vision planner can run LLM-free	Joint MLLM–planner training with multiple auxiliary tasks	Shared scene representations and planning transformer	MLLM VQA, feature distillation, planning, and surrogate tasks
VERDI [9]	No VLM computation in the driving inference path	VLM-generated reasoning plus stage-wise alignment objectives	Intermediate perception, prediction, and planning representations	Stage-wise alignment to VLM-generated reasoning features
SimpleLLM4AD [16]	VLM remains in the inference loop	VLM-based multi-stage reasoning is integral to the method	Stage-by-stage VLM reasoning forms the driving decision chain	Graph VQA over perception, prediction, planning, and behavior
TaLS (Ours)	No LLM/injection; retained Q-Former/SLG/Fusion	Qwen2.5-1.5B + LoRA and BEV Q-Former on QA-supervised training frames	Agent queries, motion queries, and ego risk/planning query	Four-stage TD-QA with answer-end hidden-state residual injection

**Table 2:** The four-stage TD-QA chain and its corresponding driving tasks.

Stage	Query	Task
Object/Scene	Describe the objects in the current scene and their locations	Detection
Motion	Describe the motion state of each object	Motion prediction
Risk	Identify dangerous situations or unsafe actions for the ego vehicle	Risk assessment
Planning	The ego vehicle’s safe driving behavior	Planning

**Table 3:** Open-loop planning results on the nuScenes validation set under the TemAvg protocol (primary), which averages the metric over all waypoints up to the N-s horizon. L2 in m and collision rate in %; lower is better. Numbers for prior methods are quoted from the corresponding papers, whereas the GenAD baseline was trained and evaluated in the same environment as TaLS; the controlled comparison is therefore between the GenAD baseline and TaLS, and its TemAvg averages match the values reported in [3]. For VLM-AD, we report the VAD-Tiny-based Q1 + Q2 configuration. Bold marks the best value in each column; ties are left unmarked.

Method	L2 (m)				Collision (%)			
	1 s	2 s	3 s	Avg.	1 s	2 s	3 s	Avg.
VAD-Tiny [2]	0.46	0.76	1.12	0.78	0.21	0.35	0.58	0.38
VAD-Base [2]	0.41	0.70	1.05	0.72	0.07	0.17	0.41	0.22
VLP-VAD [6]	0.30	0.53	0.84	0.55	**0.01**	**0.07**	0.38	0.15
VLM-AD [7]	0.30	0.54	0.80	0.55	0.11	0.15	0.38	0.21
GenAD [3]	0.27	0.49	0.81	0.52	0.14	0.18	0.25	0.19
TaLS (Ours)	0.27	0.49	0.80	0.52	0.06	0.12	**0.21**	**0.13**

**Table 4:** Open-loop planning results on the nuScenes validation set under the NoAvg protocol (secondary), which reports the value at the N-s time point alone. Conventions and sources are as in Table 3.

Method	L2 (m)				Collision (%)			
	1 s	2 s	3 s	Avg.	1 s	2 s	3 s	Avg.
ST-P3 [14]	1.33	2.11	2.90	2.11	0.23	0.62	1.27	0.71
UniAD [1]	0.48	0.96	1.65	1.03	0.05	**0.17**	0.71	0.31
VAD-Tiny [2]	0.60	1.23	2.06	1.30	0.31	0.53	1.33	0.72
VAD-Base [2]	0.54	1.15	1.98	1.22	**0.04**	0.39	1.17	0.53
GenAD	0.35	0.86	1.67	0.96	0.14	0.29	0.53	0.32
TaLS (Ours)	0.35	**0.85**	**1.64**	**0.95**	0.06	0.29	**0.47**	**0.27**

**Table 5:** Perception results on nuScenes (val). Higher is better; best in **bold**.

Method	Detection mAP	NDS	Map mAP
GenAD	0.280	0.399	**0.477**
TaLS	**0.281**	**0.400**	0.476

**Table 6:** Inference efficiency on a single NVIDIA A100-SXM4-80GB (batch size 1, FP32, six-camera nuScenes input of 1 × 6 × 3 × 384 × 640; data loading, metric computation, result serialization, and host-to-device transfer excluded). Because whole-model tracing is not supported for this architecture, FLOPs are obtained by profiling the backbone, neck, and head on their recorded inputs and summing; the decoder attention modules could not be traced and are excluded identically for both models. Overhead values are computed from the unrounded measurements.

Method	Active Params (M)	Estimated FLOPs (G)	Latency (ms)	FPS	Peak GPU Mem. (GiB)
GenAD	42.0	198.4	57.7 ± 5.3	17.4	0.52
TaLS	143.9	206.4	64.0 ± 3.8	15.6	1.22
Overhead	+101.9 (+242.6%)	+7.9 (+4.0%)	+6.3 (+10.9%)	−1.7 (−9.9%)	+0.69 (+132.3%)

**Table 7:** Prune ablation on the trained model (nuScenes val), primary TemAvg protocol. Lower is better; best in **bold**.

Method	L2 (m)				Collision (%)			
	1 s	2 s	3 s	Avg.	1 s	2 s	3 s	Avg.
GenAD	0.265	0.488	0.808	0.520	0.137	0.181	0.254	0.190
TaLS (pruned)	0.282	0.514	0.837	0.544	0.107	0.147	0.228	0.161
TaLS	0.265	**0.487**	**0.800**	**0.517**	**0.059**	**0.122**	**0.212**	**0.131**

**Table 8:** Ablation of language supervision on nuScenes (val). All TaLS ablation variants use the same data, initialization, total training budget, and compute environment; component-specific training differences are described above. GenAD is included as the baseline. Results are reported under the primary TemAvg protocol; collision rate in %, lower is better.

Method	L2 (m)				Collision (%)			
	1 s	2 s	3 s	Avg.	1 s	2 s	3 s	Avg.
GenAD	0.265	0.488	0.808	0.520	0.137	0.181	0.254	0.190
TaLS w/o language	**0.262**	**0.484**	0.805	0.517	0.098	0.156	0.270	0.175
TaLS w/o decomposition	0.267	0.490	0.802	0.520	0.078	0.132	0.221	0.144
TaLS	0.265	0.487	**0.800**	0.517	**0.059**	**0.122**	**0.212**	**0.131**

**Table 9:** Training-procedure ablation on nuScenes (val), primary TemAvg protocol. The single-stage variant is architecturally identical to the full model and uses the same 20-epoch budget and module-wise learning rates chosen to match the curriculum’s nominal scales, but is trained in a single joint phase. Collision rate in %; lower is better.

Method	L2 (m)				Collision (%)			
	1 s	2 s	3 s	Avg.	1 s	2 s	3 s	Avg.
GenAD	0.265	0.488	0.808	0.520	0.137	0.181	0.254	0.190
TaLS (single-stage)	**0.259**	**0.476**	**0.788**	**0.508**	0.273	0.303	0.436	0.338
TaLS (3-phase)	0.265	0.487	0.800	0.517	**0.059**	**0.122**	**0.212**	**0.131**

**Table 10:** Controlled ablation of stage-wise injection and auxiliary distillation on the nuScenes validation split (primary TemAvg protocol). All variants share the same Phase-1 checkpoint, initialization, training schedule, evaluation protocol, and non-ablated components. “On” denotes that the component/objective is retained and active in its scheduled training phase(s). L2 in m and collision rate in %; lower is better.

Variant	Injection	ℒ_LM_	ℒ_dist_	L2 (m)	Collision (%)
TaLS	On	On	On	0.517	0.131
TaLS w/o injection	Off	On	On	0.512	0.205
TaLS w/o distillation	On	On	Off	0.519	0.117

**Table 11:** Zero-shot robustness under fixed visual-degradation settings on nuScenes validation set (TemAvg protocol). L2 in m and collision rate in %; lower is better. Both models use frozen checkpoints and FP32 inference.

Condition	GenAD		TaLS	
	L2 (m)	Collision (%)	L2 (m)	Collision (%)
Clean	0.520	0.190	0.517	0.131
Gaussian noise	0.515	0.283	0.505	0.163
Low light	0.515	0.199	0.515	0.152
Motion blur	0.532	0.382	0.523	0.177
CAM_FRONT dropout	0.715	0.239	0.706	0.199

**Table 12:** Three-seed reproducibility analysis on the nuScenes validation set. Values are reported as mean ± sample standard deviation over random seeds 0, 1, and 2; lower is better. The per-run behavior is discussed in the text to distinguish consistent per-seed trends from initialization-sensitive variation.

Method	TemAvg		NoAvg	
	L2 (m)	Collision (%)	L2 (m)	Collision (%)
GenAD	0.542 ± 0.030	0.238 ± 0.053	0.978 ± 0.035	0.425 ± 0.111
TaLS	0.556 ± 0.053	0.244 ± 0.131	0.992 ± 0.071	0.406 ± 0.127

**Table 13:** Dataset-wide TD-QA routing census under the frame-weighted training-time selection distribution. Percentages are obtained by exhaustively applying the fixed form-level rubric to all eligible candidates; no QA subsampling is used. “Ambiguous” denotes dual-eligible questions whose semantics are compatible with more than one neighboring stage.

Stage	Frames	Clear	Ambiguous	Incorrect
Object/Scene	4072	42.2%	41.8%	16.1%
Motion	4072	31.4%	49.8%	18.8%
Risk	4072	100.0%	0.0%	0.0%
Planning	4072	37.5%	37.6%	24.9%
**ALL**	**16,288**	**52.8%**	**32.3%**	**14.9%**

**Table 14:** Fixed stage-mismatch alignment controls on nuScenes validation set. The aligned row uses the standard TaLS reference result. Mismatch A and Mismatch B apply two distinct fixed non-aligned source-to-destination permutations while retaining the remaining training objectives and schedule.

Variant	TemAvg		NoAvg	
	L2 (m)	Collision (%)	L2 (m)	Collision (%)
TaLS	0.517	0.131	0.947	0.274
TaLS (Mismatch A)	0.517	0.141	0.944	0.352
TaLS (Mismatch B)	0.514	0.141	0.941	0.273

## Data Availability

The nuScenes dataset used in this study is publicly available from the nuScenes website upon registration. The DriveLM annotations used for language supervision are available from the official DriveLM project repository, subject to their respective licenses.

## Appendix A

Table A1 lists four complete four-stage TD-QA examples from the DriveLM-annotated training split, selected by the keyword rules of Section 3.3. Examples C and D illustrate incorrect and ambiguous routing cases, respectively.

**Table A1:** Complete four-stage TD-QA examples from four training frames. <c1,CAM,…> denotes a DriveLM object reference (object identifier, camera, and image coordinates). † Incorrect routing: a scene-importance question is assigned to the Motion stage because it contains the incidental keyword “driving”, although Object/Scene is the better primary semantic match. ‡ Ambiguous routing: the Object/Scene question identifies an object by direction, which matches the Object/Scene definition, but its answer is a motion state, which matches the Motion definition equally.

Frame	Stage	Question (DriveLM)	Answer (Training Target)
A	Object/Scene	What are objects to the front left of the ego car?	There are many barriers, one pedestrian, one car, and one traffic cone to the front left of the ego car.
	Motion	What is the moving status of object <c1,CAM_FRONT,714.3,503.6>?	Going ahead.
	Risk	What actions taken by the ego vehicle can lead to a collision with <c1,CAM_FRONT,714.3,503.6>?	Accelerate and go straight.
	Planning	Predict the behavior of the ego vehicle.	The ego vehicle is going straight. The ego vehicle is driving slowly.
B	Object/Scene	What are objects to the front left of the ego car?	There are many barriers, two traffic cones, many pedestrians, three cars, one bicycle, one truck, and one construction vehicle to the front left of the ego car.
	Motion	What is the moving status of object <c4,CAM_FRONT,549.2,595.0>?	Going ahead.
	Risk	What actions taken by the ego vehicle can lead to a collision with <c2,CAM_FRONT_RIGHT,1107.5,495.8>?	Sharp right turn.
	Planning	Predict the behavior of the ego vehicle.	The ego vehicle is slightly steering to the right. The ego vehicle is driving slowly.
C	Object/Scene	What are objects to the front right of the ego car?	There is one truck and many cars to the front right of the ego car.
	Motion †	What are the important objects in the current scene? Those objects will be considered for the future reasoning and driving decision.	There is a red pickup truck to the back right of the ego vehicle, a black SUV to the front of the ego vehicle, a white car to the back of the ego vehicle, and a white truck to the front of the ego vehicle. The IDs of these objects are <c1,CAM_BACK_RIGHT,1007.5,628.3>, <c2,CAM_FRONT,668.3,489.2>, <c3,CAM_BACK,1080.0,507.5>, and <c4,CAM_FRONT,276.7,435.0>.
	Risk	What actions taken by the ego vehicle can lead to a collision with <c4,CAM_FRONT,276.7,435.0>?	Sharp left turn.
	Planning	Predict the behavior of the ego vehicle.	The ego vehicle is going straight. The ego vehicle is driving very fast.
D	Object/Scene ‡	What is the status of the bus that is to the front right of the ego car?	One bus is moving.
	Motion	What is the moving status of object <c2,CAM_FRONT,686.7,496.7>?	Going ahead.
	Risk	What actions taken by the ego vehicle can lead to a collision with <c4,CAM_FRONT_RIGHT,1049.2,558.3>?	Sharp right turn.
	Planning	Predict the behavior of the ego vehicle.	The ego vehicle is going straight. The ego vehicle is driving fast.
